# Peak glacier extinction in the mid-twenty-first century

**DOI:** 10.1038/s41558-025-02513-9

**Published:** 2025-12-15

**Authors:** Lander Van Tricht, Harry Zekollari, Matthias Huss, David R. Rounce, Lilian Schuster, Rodrigo Aguayo, Patrick Schmitt, Fabien Maussion, Brandon Tober, Daniel Farinotti

**Affiliations:** 1 Laboratory of Hydraulics, Hydrology and Glaciology (VAW), ETH Zürich, Zurich, Switzerland; 2 Swiss Federal Institute for Forest, Snow and Landscape Research (WSL), Sion, Switzerland; 3 Department of Water and Climate, Vrije Universiteit Brussel, Brussels, Belgium; 4 Department of Geosciences, University of Fribourg, Fribourg, Switzerland; 5 Department of Civil and Environmental Engineering, Carnegie Mellon University, Pittsburgh, PA USA; 6 Department of Atmospheric and Cryospheric Sciences (ACINN), Universität Innsbruck, Innsbruck, Austria; 7 Bristol Glaciology Centre, School of Geographical Sciences, University of Bristol, Bristol, UK

**Keywords:** Cryospheric science, Environmental sciences

## Abstract

Projections of glacier change typically focus on mass and area loss, yet the disappearance of individual glaciers directly threatens culturally, spiritually and touristically significant landscapes. Here, using three global glacier models, we project a sharp rise in the number of glaciers disappearing worldwide, peaking between 2041 and 2055 with up to ~4,000 glaciers vanishing annually. Regional variability reflects differences in average glacier size, local climate, the magnitude of warming and inventory completeness.

## Abstract (web summary)

Many mountain glaciers will disappear with warming. Here the authors assess how many glaciers will disappear per year under different warming scenarios, finding that a peak in glacier loss will happen during the mid-twenty-first century.

## Main

Glaciers worldwide are retreating rapidly^1,2^, with losses projected to continue throughout the twenty-first century^3,4^ and beyond^5^. Observations and projections assessed by the IPCC^6^ have primarily focused on changes in glacier mass and area, particularly in relation to sea-level rise^7^ and water availability^8^. In contrast, the evolution of the total number of glaciers has received comparatively little attention^3^, partly because glacier number is a less clearly defined metric and is influenced by observational limitations^9^. Yet knowing where and when individual glaciers will disappear is important from touristic, cultural and spiritual perspectives.

Glaciers attract millions of visitors each year, offering opportunities for recreation, education and outreach^10–12^. Many ski resorts also depend on glaciers, meaning their disappearance could affect winter tourism economies^13,14^. Beyond recreation, glaciers hold deep cultural, historical and symbolic importance. In many regions, they are iconic features tied to local traditions, spiritual practices and communal identity^15–18^. Across cultures, glaciers have also inspired stories, rituals and legends^19,20^. At the same time, even small glaciers can provide essential meltwater for communities and livelihoods, adding another dimension to their societal importance.

As glaciers shrink, communities are confronted with these changes, sometimes marking their loss with symbolic rituals, such as the ‘glacier funerals’ for Okjökull glacier (Iceland, 2019)^21^, Pizol glacier (Switzerland, 2019) (ref. ^22^) and Yala glacier (Nepal, 2025) (ref. ^23^). These ceremonies highlight the emotional and societal dimensions of glacier loss^20,23–25^. Iceland has even established a global glacier graveyard^26^, while initiatives such as the Global Glacier Casualty List aim to preserve the names and histories of vanishing glaciers^21^.

Here we offer a distinct perspective on glacier loss by quantifying the disappearance of each of the world’s more than 200,000 glaciers included in the global Randolph Glacier Inventory version 6.0 (RGI v.6.0)^27^, under four policy-relevant global warming scenarios by 2100 (relative to pre-industrial levels): +1.5 °C and +2.0 °C (Paris Agreement targets), +2.7 °C (current policy pledges) and +4.0 °C (a higher-emission pathway). Using three global glacier models, we introduce the concept of peak glacier extinction—the year in which the largest number of glaciers is projected to disappear between now (2025) and the end of the century. In this study, a glacier is classified as disappeared, or extinct, when either its projected area falls below 0.01 km^2^ (the standard inventory threshold^9^) or its remaining volume declines to less than 1% of its initial value. While this number-based framing provides an intuitive and complementary perspective to traditional mass loss metrics, it must be interpreted with care. Glacier number is sensitive to the inventories, which vary in resolution, completeness and treatment of small or fragmented ice bodies.

**Fig. 1:**
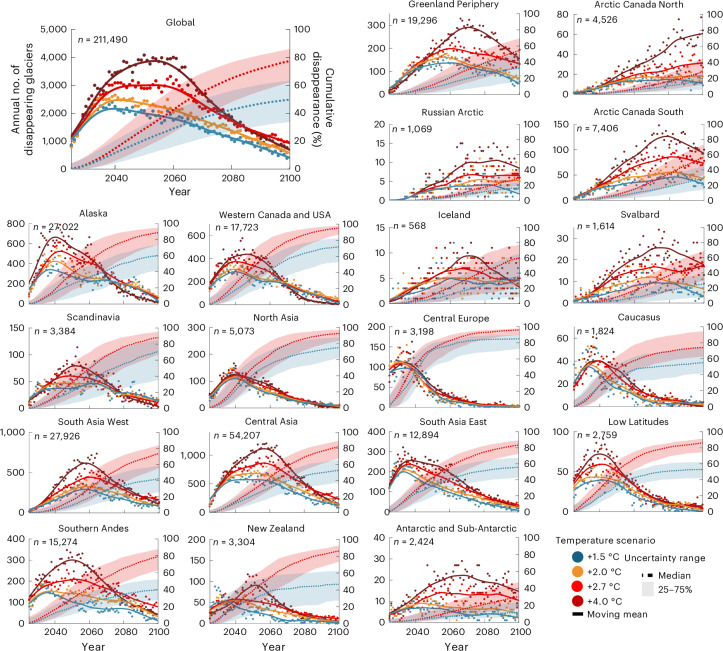
Projected annual glacier disappearance at global and regional scales under different warming scenarios. The points show the projected number of glaciers lost each year (values on the left *y* axis), according to the median year-scenario across all glacier and climate model combinations (Methods). The solid lines represent 11-year running means. The dotted lines indicate the cumulative percentage of glaciers lost since 2025 (values on the right *y* axis), with shaded bands showing the interquartile range (25th–75th percentiles). For clarity, shaded areas are shown only for the +1.5 °C and +2.7 °C scenarios. *n* indicates the total number of glaciers in 2025.

Our results show that glacier extinction (the number of individual glaciers disappearing) will peak around mid-century, with both its timing and magnitude depending on the warming level. Under +1.5 °C, global peak glacier extinction is projected to reach ~2,000 glaciers per year around 2041 (Fig. 1). Under +4.0 °C, this peak shifts to the mid-2050s and intensifies to ~4,000 per year. This delayed peak under higher warming reflects the associated longer and stronger glacier area and volume loss.

**Fig. 2:**
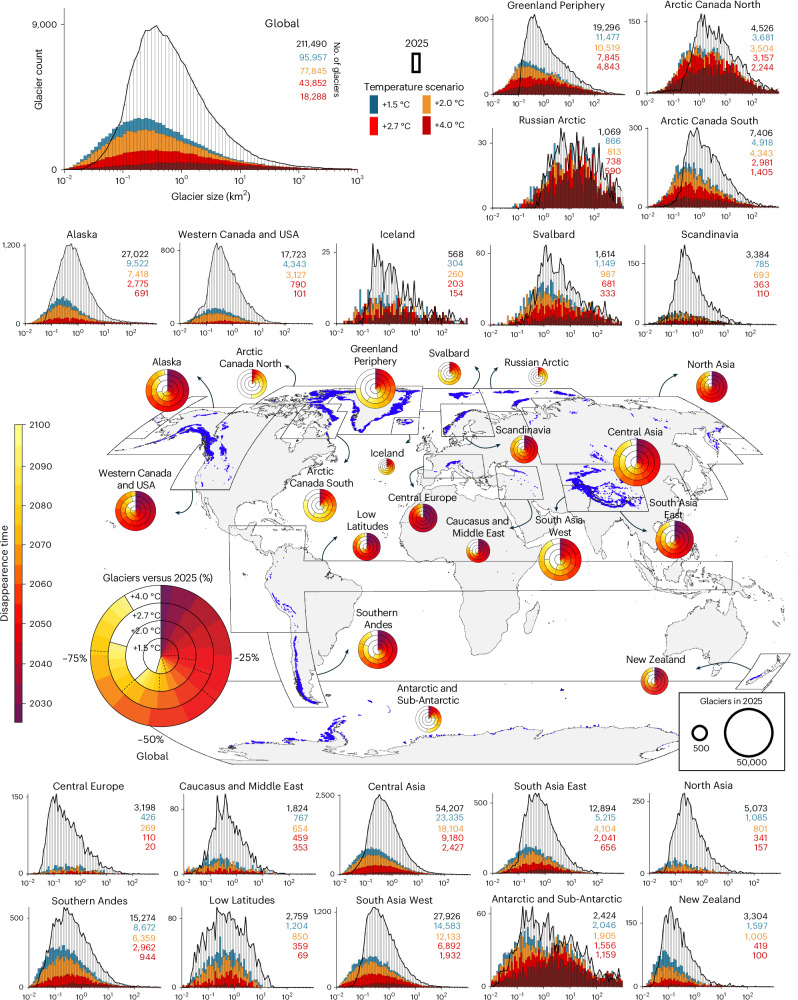
Glacier extinction in the twenty-first century. The pie charts show the number of glaciers that are expected to disappear in each global region. The colour gradient indicates when this loss is projected to occur: red hues denote earlier loss, and yellow hues represent later loss. The dashed lines mark the years 2050 and 2075. For clarity, the colours are grouped into five-year intervals. In the central world map, glacier locations at the inventory date are shown in blue. The histograms around the world map compare the number and size of glaciers in 2025 (white bars) with projections for the year 2100 under the same four global warming levels (coloured bars). The height of each bar indicates the total number of glaciers within the corresponding size interval. A solid black line highlights the height of the white bars, representing the present-day distribution. The numbers of glaciers in 2025 (black font) and 2100 (Shared Socio-economic Pathway (SSP) colours) under different warming levels are listed to the top right of each histogram. Basemap from Natural Earth (https://www.naturalearthdata.com) with glacier outlines from RGI v.6.0 (ref. ^27^).

**Extended Data Fig. 1:**
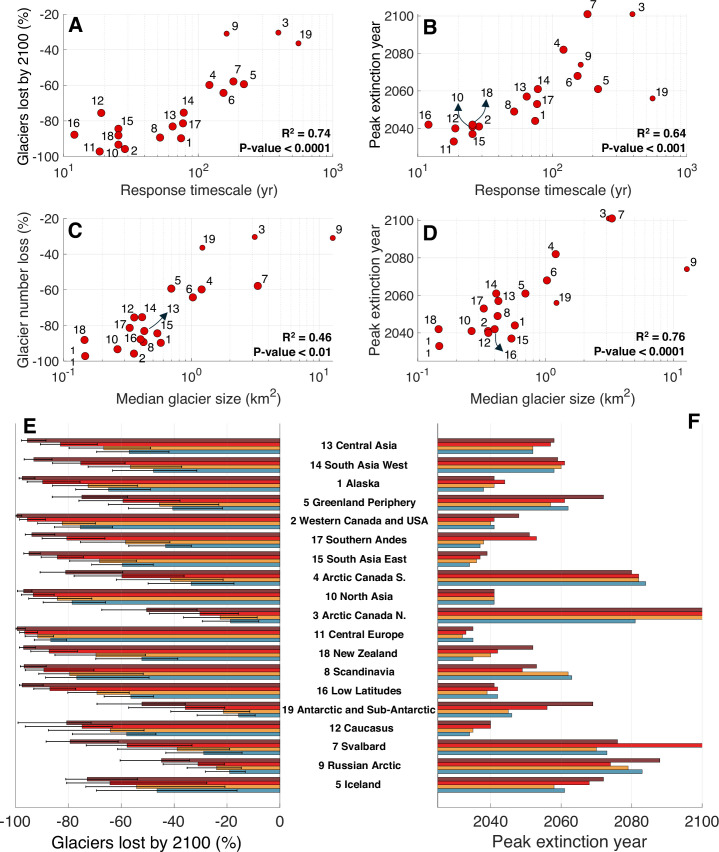
Glacier loss and peak extinction timing in relation to climate response characteristics. Panels **A** and **B** show, respectively, the number of disappearing glaciers (%) under +2.7 °C warming and the timing of peak glacier extinction (averaged across warming levels) as a function of glacier response time, defined as the number of years required to reach 80% of committed mass loss under a + 3 °C scenario^28^. Panels **C** and **D** relate glacier number loss and peak extinction year to median glacier size. Response timescales and glacier sizes are shown on a logarithmic x-axis. R² values are calculated for linear regression excluding Arctic Canada North (region 3), Russian Arctic (9), and Antarctic/Sub-Antarctic (19), which do not exhibit a clear extinction peak and lose only 30–36% of glaciers by 2100. Panels **E** and **F** show, respectively, the number of glaciers lost and the year of peak extinction across regions and warming levels for the median projection. Error bars in panel **C** denote the 25th–75th percentile range. Regions are ordered by total glacier count from high to low.

The timing of peak glacier extinction varies markedly across regions, reflecting differences in glacier size distributions (Fig. 2), inventories and response times (Extended Data Fig. 1). In regions dominated by small and rapidly responding glaciers, such as the Caucasus, the Subtropical Andes (Low Latitudes), North Asia and the European Alps (Central Europe), over 50% of the glaciers are projected to disappear within the next two decades (Figs. 1 and 2). As a result, peak glacier extinction in these regions occurs early, typically before or around 2040, and is largely insensitive to the warming level. The glaciers lost during this period are predominantly small and contribute minimally to the region’s total ice volume and area. In contrast, regions with a higher proportion of larger glaciers, including the ice sheet peripheries (Greenland and Antarctic/Sub-Antarctic), Svalbard and the Russian Arctic, exhibit a delayed peak in glacier extinction that occurs later in the twenty-first century or potentially even beyond in some regions such as Arctic Canada North. This latter region combines a large median glacier size with long response timescales (Extended Data Fig. 1). In these regions, the timing of peak extinction is also more sensitive to the warming level (Fig. 1), consistent with their slower dynamic responses and dominance of larger glaciers (Fig. 2). In some cases, it may also reflect inventory limitations, as small glaciers that are abundant in regions such as the Alps may be underrepresented in other regions^9^, delaying the apparent timing of peak extinction. High-mountain Asia hosts more than one third of all glaciers around the globe (~90,000 glaciers out of ~210,000 (ref. ^27^)), making it a key contributor to the global glacier distribution (Fig. 2). Due to the predominance of glaciers with intermediate sizes (Fig. 2), this region exhibits a distinct mid-century peak in glacier extinction, which is strongly reflected in the global pattern.

At peak glacier extinction, between ~2,000 individual glaciers per year under +1.5 °C warming and ~4,000 per year under +4.0 °C are projected to disappear globally (Fig. 1). This rate is equivalent to losing the entire glacier population of the European Alps in just one year and represents a rate three to five times higher than the present-day modelled global loss of 750–800 glaciers annually (Fig. 1). Following this peak, the annual loss rate gradually declines, reaching 700–1,200 glaciers per year by the end of the century. However, this does not mark the end of glacier disappearance: substantial glacier mass loss is expected to continue beyond 2100^5,28^, suggesting that many additional glaciers will disappear in the twenty-second century.

Regionally, maximum glacier extinction rates are influenced by both the present-day glacier number, particularly the abundance of small glaciers, and the warming level. In regions with relatively few glaciers, such as Iceland, peak annual loss remains limited to 5–10 glaciers. In contrast, Central Asia, which hosts the largest glacier population, currently loses 200–300 glaciers per year. This rate is projected to peak at ~500 per year under +1.5 °C warming, increasing to ~1,100 per year under +4.0 °C (Fig. 1). Expressed in relative terms, the maximum glacier extinction rates correspond to ~1.5% ± 0.7% of present-day glaciers per year in most regions, with lower values in high-latitude regions (for example, Antarctic and Sub-Antarctic, ~0.5%) and higher values in regions with many small glaciers (for example, Central Europe, ~3.3%).

While glaciers are expected to shrink significantly this century, many could still survive, especially if global warming is limited (Fig. 2). Under a +1.5 °C scenario, the projected number of glaciers lost over the next 20–30 years is roughly half of that expected under a +4.0 °C trajectory. By 2100, nearly 50% of today’s glaciers may remain (Figs. 1 and 2). In contrast, under current climate pledges (+2.7 °C of warming by 2100 (ref. ^29^)), annual glacier losses remain for a long time about 1,000 glaciers per year higher than for +1.5 °C warming, but lower than under the +4.0 °C pathway until around 2070. Beyond that point, glacier loss rates under +2.7 °C converge with those under +4.0 °C, ultimately leaving only ~20% of the initial glacier count by 2100 (Fig. 1). Under +4.0 °C, extinction rates remain high for decades, with 3,000 to 4,000 glaciers disappearing annually between 2035 and 2065. Fewer than 10% of present-day glaciers are projected to survive by 2100, even though larger glaciers may split up into several smaller ones while retreating (Fig. 1). These differences highlight the importance of mitigation: limiting warming to +1.5 °C could more than double the number of glaciers surviving by 2100 compared with +2.7 °C and prevent the near-complete loss expected under +4.0 °C warming, where fewer than 20,000 glaciers are projected to persist (Fig. 1 and Extended Data Table 1).

Under high warming, many regions are expected to become nearly glacier-free by 2100 (Fig. 2). For example, under +4.0 °C, only ~20 glaciers are projected to remain in Central Europe, a 99% reduction compared with the present day (Fig. 1 and Extended Data Table 1). Similar losses are expected in Western Canada and USA and in the Low Latitudes. In contrast, regions such as the Antarctic and Sub-Antarctic are projected to retain 47–64% of their glaciers under +4.0 °C and +2.7 °C warming, and up to 84% under +1.5 °C. Other regions, including Central Asia and Alaska, show a steep increase in glacier disappearance with higher warming, losing two to three times more glaciers at the peak extinction year under +4 °C than under +1.5 °C. These contrasts highlight how ambitious climate policy can make a substantial difference in preserving glaciers.

**Extended Data Fig. 2:**
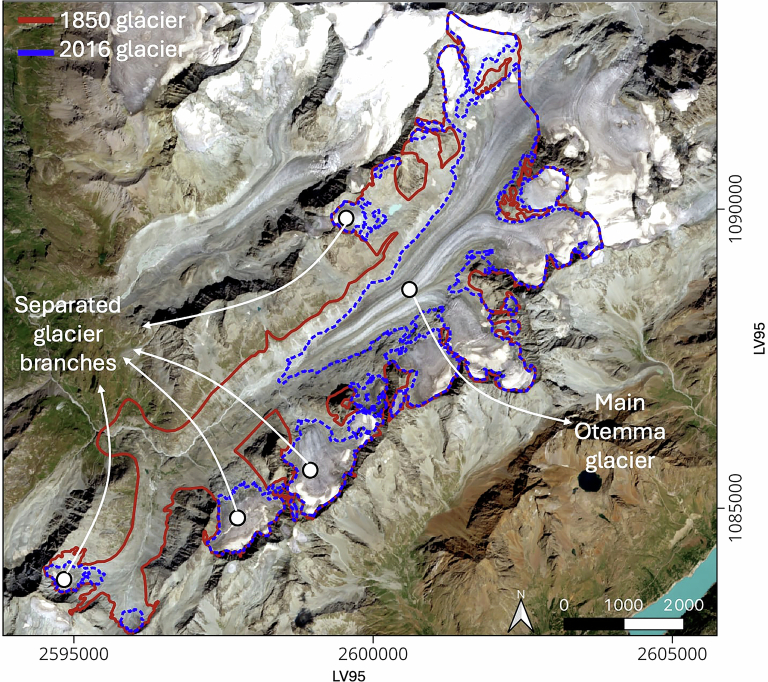
Fragmentation of Otemma Glacier, Switzerland, between 1850 and 2016. Otemma Glacier (southwestern Switzerland) has undergone significant retreat and fragmentation since the end of the Little Ice Age. A once-continuous glacier tongue has split into smaller branches, now appearing as separate ice bodies. In this study, all glacier fragments within the original outline are treated as a single glacier entity, consistent with RGI v6.0 (circa 2000). Satellite data from Copernicus (https://browser.dataspace.copernicus.eu/). 1850 outline from ref. ^44^ and 2016 outline from ref. ^45^.

Although many glaciers are projected to persist through the end of the century, their number, size and distribution will vary substantially by region, initial glacier number and warming level (Fig. 2). Our results point to a systematic shift in the glacier size distribution, with an increasing dominance of very small glaciers and a decline in larger ones (Fig. 2). This trend primarily reflects the continued shrinkage of today’s larger glaciers, which, despite substantial mass loss, are still projected to survive by 2100 in diminished form. This may, in part, also reflect limitations in current inventories, which often struggle to detect and delineate small or debris-covered ice bodies and apply varying size thresholds^9^. Small glaciers are well captured in some regions but underrepresented in others, introducing inconsistencies in counts, and influencing both extinction timing and totals. The omission of glacierets and other very small ice bodies results in wide-ranging estimates of global glacier numbers, with a central estimate of ~435,000 (ref. ^9^). While this uncertainty influences our results, it predominantly concerns very small, minimally dynamic glaciers, which are expected to disappear rapidly. With our approach, we are confident in having a very comprehensive image of the number of glaciers that currently have a decent size (exceeding a threshold of 0.01 km^2^). Additionally, current glacier models do not simulate fragmentation. Each glacier evolves as a single unit, and the formation of smaller glaciers through splitting is not accounted for (Extended Data Fig. 2). Our analysis therefore tracks the number of disappearing glaciers as inventoried around the year 2000. Given that glacier number is shaped by observational limitations and subjective classification choices, it should be interpreted with greater caution than glacier area or mass, which are more directly and objectively measurable.

The introduction of peak glacier extinction reframes the narrative of glacier change, offering a metric that complements traditional measures of glacier mass and area loss. This peak loss of individual glaciers is more than a numerical milestone: it marks a turning point with profound implications for ecosystems, water resources and cultural heritage. As mourning rituals, memorials and glacier graveyards emerge, glacier loss turns from a scientific concern into a human story of vanishing landscapes, fading traditions and disrupted daily routines. This transition also highlights the urgent need for adaptation, particularly in regions dependent on meltwater from small glaciers, which are often the first to disappear.

In the context of the UN’s International Year of Glacier Preservation 2025 and the UN Decade of Action for Cryospheric Sciences 2025–2034, our results underscore the urgency of ambitious climate policy. An earlier peak in glacier extinction, as seen under low-warming scenarios, does not mean that losses are harmless but that fewer glaciers vanish than in a later and larger peak under high-warming scenarios. An earlier peak thus reflects a more optimistic trajectory. The timeline of peak glacier extinction is not yet decided. The difference between losing 2,000 and 4,000 glaciers per year by the middle of the century is determined by near-term policies and societal decisions taken today.

## Methods

### Glacier inventory

All projections presented in this study are based on RGI v.6.0 (ref. ^27^), which provides a globally comprehensive dataset of glacier outlines. RGI v.6.0 represents a nominal snapshot around the year 2000. The inventory includes 215,543 glaciers, covering a total glacierized area of 705,253 km^2^, including those on the peripheries of Greenland and Antarctica. These glaciers collectively store an estimated 158 ± 41 × 10^3^ km^3^ of ice^30^. We used RGI v.6.0 instead of the newer RGI v.7.0 to ensure consistency with the glacier evolution models used in this study, all of which are calibrated against geodetic mass balance data^31^, and geometric input datasets specifically prepared for RGI v.6.0. The actual number of glaciers globally may be substantially higher than reported in RGI v.6.0. While it is difficult to determine the exact number of uncharted glaciers, particularly in remote regions, estimates suggest that thousands of additional, often small, glaciers may be missing from inventories due to limitations in remote sensing and differences in the definition of what constitutes a glacier. In this study, we treated each glacier entity as defined in RGI v.6.0. Statistical approaches applied in RGI v.7.0 indicate that, depending on definitions and assumptions, the total number of glaciers worldwide (including very small ones) could reach a central estimate of ~435,000.

### Climate forcing and warming levels

We used simulations of individual glacier area and volume from 2000 to 2100 using climate projections from SSPs for general climate models (GCMs) of the Coupled Model Intercomparison Project Phase 6 (CMIP6) and the three global glacier models. All three models use the same set of GCM/SSP inputs to simulate the evolution of glaciers globally (Extended Data Table 2). The selected GCMs were chosen to capture a broad range of climate sensitivities and to ensure the availability of both temperature and precipitation variables under different SSP scenarios. These GCMs were also used in previous studies^3,4^, providing continuity with previous work. Although a larger ensemble of GCMs could potentially capture a broader range of future climate outcomes, many other CMIP6 models lacked complete simulations (that is, forcing variables across all SSPs) for all three glacier models, limiting their suitability for inclusion in a consistent model intercomparison framework. To explore specific levels of global warming, we selected scenarios that yield a mean or median global temperature increase by 2071–2100 (relative to 1850–1900) near +1.5 °C, +2.0 °C, +2.7 °C and +4.0 °C. We accounted for an observed warming of 0.69 °C from 1850–1900 to 1986–2005, following IPCC AR6 guidelines^6^.

### Global glacier modelling

We used output from three global glacier models: the Global Glacier Evolution Model (GloGEM)^32^, the Open Global Glacier Model (OGGM)^33^ and the Python Glacier Evolution Model (PyGEM)^34^. These models simulate annual glacier area and volume changes from the RGI reference year to 2100 using a one-dimensional flowline approach, in which each glacier is represented by a flowline capturing simplified ice dynamics. At each time step, ice thickness is updated on the basis of a combination of surface mass balance and ice flow. The simulations used here were previously used for assessments of global glacier mass and area changes and sea-level contributions^4^. In this study, we reanalysed those simulations to assess the disappearance of individual glaciers on the basis of their area and volume trajectories. GloGEM^32^, OGGM^33^ and PyGEM^34^ provide complementary frameworks for simulating global glacier evolution, each combining surface mass balance and ice dynamics in distinct ways. The three models use a temperature-index model to compute mass balance on the basis of precipitation, temperature and refreezing. In GloGEM, calibration follows a three-step procedure that adjusts precipitation, melt factors and temperature offsets, with iterative tuning. OGGM starts with a similar three-step procedure to calibrate mass balance and then refines the melt factor using a dynamic spin-up method^4,35^, while PyGEM uses a Bayesian approach with Gaussian process emulators to efficiently capture glacier-specific sensitivities. In GloGEM, the glacier geometry is updated annually via an empirical hypsometric retreat parameterization. OGGM and PyGEM employ a one-dimensional, depth-integrated ice dynamics model that solves the mass continuity equation along elevation band flowlines. Ice viscosity is calibrated regionally to match the consensus ice thickness estimate during glacier bed inversion. Currently, none of these models incorporate time-evolving ice temperatures to modulate viscosity. While thermomechanical couplings are possible for single-glacier studies^36,37^, such approaches remain computationally infeasible at the global scale, and suitable calibration data are still limited. Future developments and new datasets^38^ may enable the implementation of simplified parameterizations linking viscosity to climatic conditions. However, although changing ice temperature may affect local glacier behaviour, its impact on large-scale projections is expected to remain limited.

### Definition of glacier disappearance

A glacier is defined as a land-based body of ice formed by the accumulation and recrystallization of snow and exhibiting evidence of past or present flow. Following RGI v.6.0 and World Glacier Inventory conventions^9^, we adopted a minimum glacier area of 0.01 km^2^ (1 hectare). However, not all regional inventories within RGI v.6.0 apply this threshold uniformly, with some using slightly higher size cut-offs. We classified a glacier as disappeared when either its projected area drops below 0.01 km^2^ or its remaining volume declines to less than 1% of its initial value. To reduce noise from short-term variability (for example, temporary regrowth during cold years), we applied a five-year Gaussian filter to smooth the glacier area and volume time series. The volume threshold complements the area criterion by capturing the loss of dynamically meaningful ice. Our approach is more conservative than that used by the Goodbye Glaciers project (https://goodbye-glaciers.info/glaciers/), which defines disappearance at a 10% volume threshold. Glaciers are considered disappeared in the first year that either criterion is met. In overshoot scenarios (for example, temporary warming beyond a long-term +1.5 °C target), regrowing glaciers are not reclassified as surviving. Importantly, our framework does not account for a potential increase in glacier count due to the fragmentation of large, branching glaciers into smaller entities during retreat. We consider any smaller sub-glaciers that may emerge as a glacier retreats and fragments (Extended Data Fig. 2) to remain part of the present-day glacier and therefore do not explicitly quantify them as new, smaller glaciers. While our approach does not explicitly account for such fragmentation, future 3D modelling techniques^39,40^ may enable this.

### Model combination

Disappearance years are identified for each glacier in the world on the basis of the thresholds described above. We report the median year across all simulations as the central estimate, along with the 25th and 75th percentiles to express uncertainty (Extended Data Fig. 1 and Extended Data Table 1). As detailed in Extended Data Table 2, the ensemble includes varying numbers of simulations per warming level depending on the available climate scenarios: 12 simulations for +1.5 °C (3 glacier models × 4 scenarios), 30 for +2.0 °C (3 × 10), 42 for +2.7 °C (3 × 14) and 21 for +4.0 °C (3 × 7).

### Glacier number loss prior to 2025

Our simulations also account for glaciers projected to have disappeared between the RGI reference year (~2000) and the start of our analysis window (2025). These early losses primarily affect very small glaciers, especially in regions such as Western Canada and USA, the Low Latitudes and Central Europe. Model output suggests that 6%, 7% and 19% of glaciers in these regions, respectively, have already disappeared. These areas also correspond to the most negative observed glacier mass balances globally over the 2000–2023 period^1,2^. In regions such as Central Europe, where peak extinction is projected to occur soon after 2025, the maximum rate of glacier loss may have already passed, although this cannot yet be confirmed observationally. It is important to note that all the numbers we report refer to the number of modelled glaciers in 2025. As such, the number of initial glaciers differs from the original numbers as given by the inventory^27^.

### Regional and global glacier extinction

After assigning a median disappearance year to each glacier or identifying those projected to persist beyond 2100, we calculated the number of glacier disappearances per year at global and regional scales. These annual totals are shown as scatter points in Fig. 1. To reduce interannual variability, we applied an 11-year Gaussian moving average. This longer smoothing window is consistent with conventions used in climate-related time series such as in global temperature trends, radiative forcing estimates and peak water timing. It allows us to better highlight multi-decadal patterns in extinction rates, which are more meaningful for interpreting long-term trends. The year when this smoothed curve reaches its maximum defines the peak glacier extinction year. Another widely used glacier-change metric is peak water^41^, the maximum meltwater release from a glacier, which always precedes extinction at the individual scale. Regionally, however, glacier extinction peaks may occur earlier in areas dominated by small glaciers, where runoff is governed by the largest ice masses. For glaciers projected to survive beyond 2100, defined as those for which at least 50% of simulations retain both area and volume above the defined thresholds, we also computed projected glacier area in 2100. These values were used to estimate future glacier size distributions, shown in the bar plots in Fig. 2.

## Online content

Any methods, additional references, Nature Portfolio reporting summaries, source data, extended data, supplementary information, acknowledgements, peer review information; details of author contributions and competing interests; and statements of data and code availability are available at 10.1038/s41558-025-02513-9.

## Data Availability

All data from the original studies^3,4^ were processed and are available together with the regional and glacier-specific results via Zenodo at 10.5281/zenodo.17371641 (ref. ^42^).

## Extended data

**Extended Data Table 1:** Glacier extinction statistics by region and globally

Region	Total glaciers in 2025	Peak extinction year (± StD)		Lost at peak year (± StD)		Remaining by 2100 (difference vs 2025)							
						+1.5 °C		+2 °C		+2.7 °C		+4 °C	
Central Asia	54 207	2056	±3	802	±236	23 335	-57%	18 104	-67%	9 180	-83%	2 427	-96%
South Asia West	27 926	2060	±1	418	±146	14 583	-48%	12 133	-57%	6 892	-75%	1 932	-93%
Alaska	27 022	2042	±2	483	±142	9 522	-65%	7 418	-73%	2 775	-90%	691	-97%
Greenland Periphery	19 295	2063	±8	188	±57	11 477	-41%	10 519	-46%	7 845	-59%	4 843	-75%
Western Canada and USA	17 723	2043	±4	346	±67	4 343	-75%	3 127	-82%	790	-96%	101	-99%
Southern Andes	15 274	2047	±8	189	±78	8 672	-43%	6 359	-58%	2 962	-81%	944	-94%
South Asia East	12 894	2037	±2	224	±28	5 215	-60%	4 104	-68%	2 041	-84%	656	-95%
Arctic Canada S.	7 406	2081	±1	78	±36	4 918	-34%	4 343	-41%	2 981	-60%	1 405	-81%
North Asia	5 073	2041	±0	118	±7	1 085	-79%	801	-84%	341	-93%	157	-97%
Arctic Canada N.	4 526	2100	±0	30	±22	3 681	-19%	3 504	-23%	3 157	-30%	2 244	-50%
Central Europe	3 198	2033	±2	106	±6	426	-87%	269	-92%	110	-97%	20	-99%
New Zealand	3 304	2044	±6	54	±17	1 579	-52%	1 005	-70%	419	-87%	100	-97%
Scandinavia	3 384	2055	±7	56	±17	785	-77%	693	-80%	363	-89%	110	-97%
Low Latitudes	2 759	2040	±2	54	±15	1 204	-56%	850	-69%	359	-87%	69	-97%
Antarctic and Sub-Antarctic	2 424	2056	±12	12	±6	2 046	-16%	1 905	-21%	1556	-36%	1 159	-52%
Caucasus	1 824	2038	±3	37	±4	767	-58%	654	-45%	459	-75%	353	-81%
Svalbard	1 614	2082	±16	15	±7	1 149	-29%	987	-39%	681	-58%	333	-79%
Russian Arctic	1 069	2080	±7	6	±3	866	-19%	813	-24%	738	-31%	590	-45%
Iceland	568	2066	±7	6	±2	304	-46%	260	-54%	203	-64%	154	-73%
**Global**	**211 490**	**2051**	**±7**	**2 792**	**±808**	**95 957**	**-55%**	**77 848**	**-63%**	**43 852**	**-79%**	**18 288**	**-91%**

Summary of glacier numbers in 2025, the peak extinction year (averaged across warming scenarios), associated average magnitude of loss at that peak, and projected number of glaciers remaining by 2100 under different warming levels (median scenario).

**Extended Data Table 2:** Climate model combinations used for each global warming level

	+1.5 °C (n = 4)		+2.0 °C (n = 10)		+2.7 °C (n = 14)		+4.0 °C (n = 7)	
**GCMs and SSPs**	GFDL-ESM4	ssp126	BCC-CSM2-MR	ssp126	BCC-CSM2-MR	ssp245	BCC-CSM2-MR	ssp370
	INM-CM4-8	ssp126	CESM2	ssp126	CESM2	ssp126	BCC-CSM2-MR	ssp585
	INM-CM5-0	ssp126	GFDL-ESM4	ssp126	CESM2	ssp245	CESM2	ssp370
	MPI-ESM1-2-HR	ssp126	INM-CM4-8	ssp126	CESM2-WACCM	ssp126	CESM2-WACCM	ssp370
			INM-CM4-8	ssp245	CESM2-WACCM	ssp245	GFDL-ESM4	ssp585
			INM-CM5-0	ssp126	GFDL-ESM4	ssp245	MPI-ESM1-2-HR	ssp585
			INM-CM5-0	ssp245	GFDL-ESM4	ssp370	MRI-ESM2-0	ssp585
			MPI-ESM1-2-HR	ssp126	INM-CM4-8	ssp245		
			MPI-ESM1-2-HR	ssp245	INM-CM4-8	ssp370		
			MRI-ESM2-0	ssp126	INM-CM5-0	ssp245		
					INM-CM5-0	ssp370		
					MPI-ESM1-2-HR	ssp245		
					MRI-ESM2-0	ssp245		
					MRI-ESM2-0	ssp370		
**Range (°C)**	**[+1.4, +1.6]**		**[+1.5, +2.4]**		**[+2.2, +3.2]**		**[+3.5, +4.5]**	
**Median (°C)**	**+1.5**		**+1.9**		**+2.7**		**+4.0**	
**Mean (°C)**	**+1.5**		**+2.0**		**+2.8**		**+4.0**	

Summary of the General Circulation Model (GCM) and Shared Socioeconomic Pathway (SSP) combinations selected for each targeted warming level. The temperature range column indicates the span of projected global mean temperature changes (2071-2100 relative to 1850-1900) across the selected scenarios. The median and mean values reflect the central tendency of temperature changes for the respective ensemble subsets. Note that the mean value of the warming level is for the +1.5 °C warming level is 1.56 °C to increase the sample size of simulations for the +1.5 °C target.

## Code availability

The code used to process the data and generate the results is available via Zenodo at 10.5281/zenodo.17416013 (ref. ^43^).
